# Polish-Lithuanian Border Cuisine as an Idea for the Promotion and Expansion of the Region’s Tourist Attractiveness

**DOI:** 10.3390/foods12132606

**Published:** 2023-07-05

**Authors:** Andrzej Soroka, Anna Mazurek-Kusiak, Szymon Chmielewski, Agnieszka Godlewska

**Affiliations:** 1 Faculty of Medical and Health Sciences, Siedlce University of Natural Sciences and Humanities, B. Prusa 14 St., 08-110 Siedlce, Poland; andrzej.soroka@uph.edu.pl; 2 Department of Tourism and Recreation, University of Life Sciences in Lublin, Akademicka 15, 20-950 Lublin, Poland; anna.mazurek@up.lublin.pl; 3 Department of Grassland and Landscape Studies, University of Life Sciences in Lublin, 20-950 Lublin, Poland; szymon.chmielewski@up.lublin.pl

**Keywords:** culinary tourism, food habits, regional distinctiveness, regional dishes, local food, regional flavours

## Abstract

Culinary tourism is one of the most rapidly developing forms of tourism in the world. The objective of this study is to evaluate the role of and tourists’ familiarity with cuisine in the area along the Polish-Lithuanian border. The survey included adult tourists visiting areas on both sides of the border between Poland (Podlaskie Voivodship) and Lithuania (Olicki District). A total of 789 questionnaires were completed, of which 759 were included in the study: 376 from Olicki District in Lithuania and 383 from Podlaskie Voivodship in Poland. The questions in the authorial questionnaire pertained to six dishes representing the most popular regional specialities in the area on both sides of the Polish-Lithuanian border. For all the responses, a five-point Likert scale was used. The results of the work show that tourists want to explore a given region by tasting local cuisine and that they are in search of unique products while paying much attention to the smell and taste of the dishes they consume. The product’s appearance is of less significance while making purchasing or consumption decisions. Flavour-related experiences associated with and preferences for individual dishes representing Polish-Lithuanian cuisine were very similar for both groups of respondents. It indicates there is a possibility of establishing cooperation to promote the products in the study area and thus enhance its tourist appeal.

## 1. Introduction

Social and cultural transformations in the modern world have been creating a new system of values which allow the development of a new lifestyle. In addition, the tourism sector is undergoing change, as there is observed constant evaluation of motives associated with tourist trips [1]. While travelling, tourists expect to undertake activities which are different from those constituting everyday responsibilities. Relaxation and the need to experience something out of the ordinary lead to pleasant new experiences [2]. To those who are partial to culinary tourism, consumption of traditional regional dishes may markedly enhance travelling experiences, which makes culinary tourism an interesting new trend. It emerged when tourists started to discover differences between food-related habits of various regions in the world [3]. At present, interest in this form of tourism is substantial for the following reasons: meals are always consumed during tourist trips; eating is one of favourite activities undertaken by tourists; regional cuisine and discovering the region’s cultural heritage are one of motives behind choosing a travel destination; it has become fashionable to get to know one’s origins and traditions; this type of tourism can be enjoyed by almost every segment of tourists; and cuisine always positively affects tourists’ feelings [4].

Culinary tourism, particularly in rural areas, involves tasting regional dishes made from products typical of a given area. Dish degustation is often accompanied by an opportunity to purchase the dish [5]. It is associated with cultural tradition, ethnography, handicraft, customs, regional history, local patois and attire [6]. By making use of cultural heritage, a region may attract tourists and increase tourist movement by preserving the type of dishes on offer, the places where they can be consumed and the positive perception of the region by tourists [7]. Carpio et al. [8] claim that culinary tourism bridges culture and food.

Tourist movement along the Polish-Lithuanian border accounts for barely 2% of the whole tourist movement in Poland and Lithuania [9]. Promotion of this region through traditional cuisine and regional dishes may be one of the ways of increasing tourist movement, and thereby the amount of money spent by tourists.

The objective of this study is to evaluate the role of and tourists’ familiarity with cuisine in the area along the Polish-Lithuanian border. The following research hypotheses were advanced:

1. Attractiveness of regional dishes, their appearance, flavour and smell affect consumers’ purchasing decisions, which contributes to the promotion of Polish-Lithuanian border regions by clearly emphasising their originality and distinctiveness;
2. Some traditional dishes offered by Polish-Lithuanian cuisine constitute the region’s tourist attraction, and, as such, they affect the region’s promotion, which results in increasing tourist arrivals in these areas of Poland and Lithuania;
3. Culinary tourism provides social, cultural and economic benefits to locals.

There was also an attempt to gain information on the effect of regional cuisine, including specific dishes, on the region’s promotion, distinctiveness and development of original tourist attractions based on the two elements.

## 2. Literature Review

According to the World Food Travel Association, culinary tourism is one of the most rapidly developing forms of tourism in the world [10], which consists of getting to know the uniqueness of available local food, regional flavours and traditional dishes cooked following recipes handed down from generation to generation [11]. Culinary tourism is one of the ways of immersing oneself in other cultures, traditions and flavours [12,13]. Its participants are people for whom consumption and sampling of dishes and beverages is an important element of spending their leisure time [14]. The requirement for culinary tourism is the preservation of high-quality dishes prepared according to the region’s culinary tradition and using organic and locally available ingredients and which are served in suitable surroundings [15].

Culinary tourism encompasses a wide range of experiences, including culinary trips/tours, cooking classes, wine tasting, tours of breweries, marketing experiences, culinary competitions, festivals, visits to food production facilities and, recently, even cooking while on holiday [16,17,18].

Previous research into culinary tourism pertained to its role as a new industry [19] or a stimulus of food industry and agriculture [20]. At present, it is one of the major factors motivating tourists to choose a destination [21] and the reason for destinations’ sustainable development [22]. Also, a cross-border aspect of services has been indicated, with suggestions they should be granted a framework related to freedom of action in European Union countries with an established system of terms and conditions of providing such services [23]. Culinary tourism is associated with regional food defined as a product from a local area [24,25,26] together with its cultural factors and original production processes [27,28,29].

The increasing wealth of central eastern European societies has contributed to an increasing numbers of citizens of these countries undertaking individual culinary travels and becoming familiar with new flavours [30]. Sampling of dishes and culinary products is becoming the main objective of such trips [31]. Tourists travel to experience unusual situations and new sensations; they want to travel ‘into the past’—go back to their childhood and get to know the culinary heritage of their neighbours. According to Gulbicka [32], culinary heritage includes food and dishes characterised by special attributes and traditional cooking and production methods which originated in the distant past and which occur in a specific geographical area. It is also defined by small-scale production using specific skills and technologies [33].

Traditional culinary products and dishes on offer play a very important role in the development of agritourism and tourism in rural areas [34]. Selling of traditional dishes increases a region’s competitiveness, diversifies income and enhances the offerings of agritourism farms [35], which contributes to increased profitability for agricultural farms and, as a result, enhances the standard of life of farmers [36]. The products often constitute the local cultural heritage, which should be protected so that it is not forgotten [37,38]. This, in turn, generates specific local solidarity among people which leads to the development of a global social space. Local cultural heritage affects the development of sustainable strategies for building European society which have to take into account forms of social support contributing to solidarity among people [39].

The above is also the case in the area on both sides of the Polish-Lithuanian border, although culinary tourism in Lithuania is taking its first steps, as is reflected in, e.g., the National Strategy of Tourism Development in Lithuania, in which no stress is put on this type of tourism [40]. What is more, there is no one clear-cut definition of what constitutes ‘Lithuanian culinary heritage’ [41].

The belt along the Polish-Lithuanian border is made up of areas in north-eastern Poland and southern Lithuania. The inhabitants of the region include Lithuanians, Poles, Ukrainians, Belarusians and Tatars [42]. Throughout centuries, these cultures, their values and their traditions blended. Common history and politics were difficult and very complicated due to the friction between the cultures of East and West, the Latin and Byzantine religions and Eastern European and Western European traditions. These are the main reasons behind the emergence of ‘border cuisine’ with its specific attributes and diverse flavours [43].

Three factors affecting the culinary identity in a given area can be distinguished: cuisine of ethnic minorities; cuisine and tradition of a nation’s majorities supported by the central government of the country where minorities live; and tourism and cultural organisations and societies which support the promotion of the region’s culinary heritage [44].

Lithuanian cuisine developed through blending of gentry and peasant cuisines. Its characteristics included a small number of soups which were replaced by various salads and dishes served cold. Also, there were a large number of meat-based dishes. Lithuania ‘taught’ Europe how to smoke and preserve food [41]. Polish cuisine in the past relied heavily on the use of salt and included consumption of a wide range of groats. It mainly consisted of plain dishes including meat of livestock, poultry and wild game, eggs, fish and various cereals. Polish dishes were very high in calories [43].

The border cuisine developed when the Jagiellonian Merchant Route was formed along the eastern part of Poland. Traditional border cuisine dishes include *kartacze*, *kiszka ziemniaczana*, *soczewiaki*, *kołduny*, *czenaki* and *chłodnik litewski*. *Kartacze* are large oval potato dumplings usually filled with meat. In Lithuania, they are called *cepeliny* (*didžkukuliai*). Every year in August, Gołdapia hosts a *kartacze* fair called the Regional Borderline Festival Kartaczewo [45]. *Kiszka ziemniaczana* is of Belarusian origin. First, a mixture of grated potatoes, bacon and onions is prepared. Then, it is put inside a pork intestine. Although very time-consuming, the dish is very tasty and full of flavour. Once baked, it looks like a sausage and is most frequently consumed directly after cooking. Uroczysko near Supraśl is the locality which in August holds the *Babka* and *Kiszka Ziemniaczana* Baking Championship [46]. *Soczewiaki*, a Lithuanian cuisine speciality, are either potato buns or large potato dumplings filled with lentils which have been fried with onion and bacon and then baked. Believed in the past to be the staple diet of poor people, they can be served as the main course or a side dish. They have a typical Podlasie flavour. The tastiest *soczewiaki* are served hot, often eaten with red borsch. *Kołduny*, usually called *uszka* (small ears) in Poland, are a typical dish of Polish, Lithuanian and Belarusian cuisines. They are small dumplings made of very delicate pastry and filled with meat stuffing. Usually, they are served in chicken soup or red borsch [47]. *Czenaki* originated from Ukraine and Georgia, and they are prepared in a lidded clay pot in which layers of potatoes, meat, seasonal vegetables and mushrooms are placed alternately. Next, vegetable, tomato or chicken stock is poured over, and the pot is placed in an oven for stewing. When ready, a spoonful of sour cream or a handful of salt-brined cucumber can be added on top. *Chłodnik litewski* is a soup prepared with red beet leaves combined with sour milk or cream. It has a very characteristic pink colour and is usually served cold in the summer [43].

The abovementioned dishes are culinary specialties and market niche products which should be utilised for promoting the areas on both sides of the Polish-Lithuanian border. On the one hand, they offer an opportunity for the development of local enterprises [48] and small communities and, on the other hand, are a great attraction for consumers and tourists [34] which only need adequate promotion [49] to encourage tourists to participate in traditional cuisine fairs and festivals.

## 3. Materials and Methods

### 3.1. Study Population and Research Procedure

A questionnaire, a research technique of diagnostic surveys, was used in the study. The diagnostic survey method is frequently used in research into the hotel and gastronomic industry, as it is a reliable technique which makes it possible to study a larger population [50,51,52]. The survey was conducted from April to September 2022 and included adult tourists visiting areas on both sides of the border between Poland (Podlaskie Voivodship) and Lithuania (Olicki District). It was conducted until the required number of questionnaires was collected.

**Figure 1:**
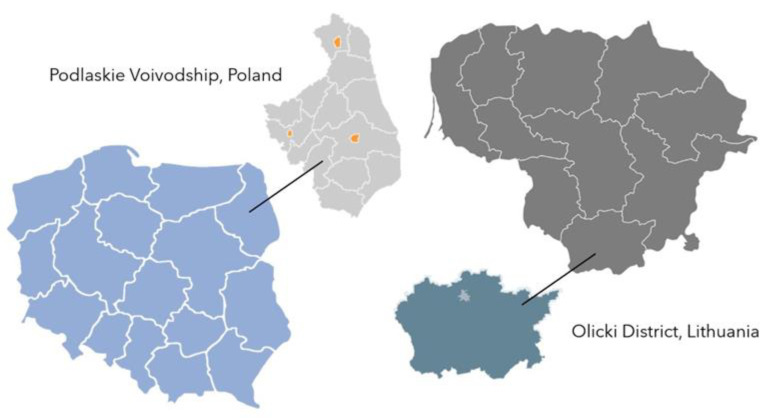
The study area—Podlaskie Voivodship, Poland; Olicki District, Lithuania. Source: Authors’ own elaboration based on the maps [53,54,55,56].

The choice of these areas was due to the Polish-Lithuanian border along which lie Podlaskie Voivodship and Olicki District (Figure 1). A total of 789 questionnaires were completed, of which 759 were included in the study: 376 from Olicki District in Lithuania and 376 from Podlaskie Voivodship in Poland. The numbers were established while deciding on the size of the research sample which was to represent the population of 18,647 tourists visiting Olicki District, and 151,779 visitors to Podlaskie Voivodship [9]. In the calculation of the research sample size, the confidence level was 95%, the fraction size was 0.5, and the maximum error was 5%.

The questions in the questionnaire pertained to six dishes representing the most popular regional specialities in the area on both sides of the Polish-Lithuanian border. The choice of dishes was made based on interviews and observations as well as the available literature [34,43,45,46,47]. The dishes were as follows: *kartacze*, *kiszka ziemniaczana*, *soczewiaki*, *kołduny*, *czenaki* and *chłodnik litewski*. In the questionnaire, the tourists visiting the study areas were asked about their familiarity with and frequency of consumption of the dishes as well as the place and circumstances of their consumption, enumerating restaurants, fairs/festivals and business meetings/conferences. Also, the respondents were asked to evaluate the visual appeal, flavour and smell of the dishes. Additionally, an attempt was made to collect information on the effect of regional cuisine, including individual dishes, on the region’s promotion, regional distinctiveness and development of original tourist attractions based on the above elements. For all the responses, a five-point Likert scale was used.

A preliminary survey including 86 people was carried out, of which 39 were visitors to the Polish region, and 47 to the Lithuanian district. The aim of this survey was to make sure the questionnaire was understood by the persons who completed it. Moreover, an initial test was conducted to validate the questionnaire which was also subjected to an internal validation adjusted to the project so as to obtain the best possible results. In the internal questionnaire validation, Cronbach’s alpha value was 0.826, which indicates the tool was reliable since the alpha was higher than the recommended value of 0.7.

The study conforms to the code of ethics of the World Medical Association and the standards for research involving human subjects set out in the Declaration of Helsinki. The protocol was approved by the local university ethics committee. This research was performed following relevant guidelines and regulations. After an explanation of the aims and protocol of this study to each participant, his/her written informed consent was obtained. The survey was conducted in a direct manner, and the participants were informed that the survey was anonymous.

### 3.2. Statistical Analysis

STATISTICA 13 PL (TIBCO Software, Palo Alto, CA, USA) software, with a discriminant function, and the Student t-test were used to conduct statistical analysis. Discriminant analysis is a statistical method used to examine differences between two groups (such as in the study reported here) with a simultaneous analysis of variables to determine which variables are the greatest contributors to group discrimination. The classification of cases is one of the main goals behind an application of discriminant analysis. The applied classification function is based on a linear combination of discriminant variables. There were two functions, the same number as the number of groups, used to determine to which group a given case belonged. Multidimensional normality was examined before testing, checking each variable for normality of distribution. We used the Kołmogorow–Smirnow test, which verified the hypothesis that two samples were taken from different populations. The arithmetic means and standard deviations were defined. When the assumptions of variables were fulfilled, the Student t-test for independent groups was used in order to define differences between mean values performance indicators. The mean differences that were defined as statistically significant were those with a probability less than *p* ≤ 0.050.

## 4. Results

Analysis of the popularity of Polish-Lithuanian cuisine with tourists visiting the area along the Polish-Lithuanian border demonstrated that *kiszka ziemniaczana* was the best-known and most frequently consumed dish. In particular for visitors to Lithuania, it reached the maximum declared mean and, at *p* ≤ 0.001, was significantly higher than the mean calculated for tourists visiting the study area in Poland. The next dishes, which had similar mean values for respondents in Poland and Lithuania, were *chłodnik litewski* and *czenaki*. *Soczewiaki* was significantly more frequently indicated by tourists in Poland, at *p* ≤ 0.001, as the dish which was more eagerly and frequently consumed compared with visitors to Lithuania. *Kartacze* and *kołduny* were similarly frequent choices made by tourists visiting both countries (Table 1).

A discriminant function model was created, and it determined a unique order of importance for the individual dishes. Of the six dishes, four entered the model, which excluded the dishes which were less important for respondents, that is, *czenaki* and *kartacze*. The highest value of classification function was obtained for *kiszka ziemniaczana*, it being significantly higher, at *p* ≤ 0.001, for respondents in Lithuania, compared with the participants in Poland. *Kołduny* was the dish with the second highest value of classification function, and both groups of tourists pointed to this dish with a similar frequency. At *p* ≤ 0.001, visitors to Lithuania significantly more often selected *soczewiaki* and *chłodnik litewski* than tourists in Poland (Table 2).

Analysis of the places where tourists consumed dishes representing Polish-Lithuanian cuisine demonstrated that *kartacze* was the dish most frequently consumed by tourists in restaurants and during fairs, whether they were visitors to Poland or Lithuania. In turn, *kiszka ziemniaczana* was frequently eaten at restaurants, this tendency being similarly frequent for respondents in Poland and Lithuania. At *p* = 0.050, tourists in Lithuania more often pointed to consumption of *soczewiaki* during conferences. Moreover, respondents in both the countries indicated conferences as events during which they consumed *chłodnik litewski*. The frequency of places of consumption of the remaining dishes was at a much lower level (Table 3).

The highest discriminatory power in the created model including places of consumption of Polish-Lithuanian dishes was displayed in the consumption of *kiszka ziemniaczana* during fairs or festivals. At *p* = 0.002, higher values of classification function were obtained for survey participants in Poland. Analysis of dishes and places of their consumption pointed to *kartacze*, which were significantly more often eaten during fairs by tourists in Lithuania. Respondents chose to eat *chłodnik litewski* and *czenaki* during conferences or business meetings. Also, fairs were places where the following dishes were the most frequently consumed: *kołduny, soczewiaki* and *chłodnik litewski*. Only for *kiszka ziemniaczana* was the restaurant the place of consumption significantly more often indicated by tourists in Poland (Table 4).

According to respondents, *kartacze*, *kołduny*, *chłodnik litewski* and *czenaki* had the most appealing appearance, with only the last dish being identified significantly more often, at *p* = 0.010, by visitors to Lithuania. Smell-related appeal was the most frequently indicated for *kołduny*, *chłodnik litewski* and *czenaki*. It should be noted that the highest mean values were reached for *kołduny*, also being significantly higher for tourists in Poland versus those in Lithuania, at *p* ≤ 0.001. As far as flavour was concerned, the highest scores were given to *czenaki*, *chłodnik litewski*, *soczewiaki* and *kołduny*. At *p* = 0.012 and *p* = 0.04, respectively, significantly more attention was paid to *chłodnik litewski* and *kołduny* by tourists visiting Lithuania. In other cases, the appearance, flavour and smell of dishes had decidedly lower mean scores given by visitors to both Poland and Lithuania (Table 5).

In the created model of discriminant function, the highest value was obtained for the smell of *kołduny*, with it being significantly more appealing for tourists in Lithuania than in Poland, at *p* ≤ 0.001, and the same was true for *soczewiaki*. The same attention was paid by respondents to the flavour and smell of *chłodnik litewski*, although tourists on the Polish side of the border pointed to the appearance of *kartacze* significantly more often, at *p* = 0.027. The flavour of *kołduny* was significantly more important, at *p* = 0.048, for tourists in Poland, whereas visitors in Lithuania indicated the flavour of *czenaki* more frequently, at *p* = 0.018. The created model included the appearance of *kołduny* as well (Table 6).

The following dishes were the most often recognised as tourist attractions for visitors in the border area: *kartacze*, *czenaki*, *kiszka ziemniaczana* and *kołduny*. At *p* ≤ 0.001 and *p* = 0.002, respectively, significantly higher mean values of scores were obtained for *kartacze* and *czenaki* evaluated by respondents in Poland and, at *p* = 0.002, for *kartacze* chosen by respondents in Lithuania. *Kołduny* and *czenaki* were considered of importance for the region’s promotion, with *czenaki* being significantly more important, at *p* = 0.024, for visitors to Lithuania. Of the six dishes subjected to investigation in the study reported here, five products were declared to be dishes specific to the region, with significant differences, at *p* ≤ 0.001, being detected for *kiszka ziemniaczana* and *chłodnik litewski*, and higher scores being granted by tourists in Lithuania (Table 7).

In the created model, *czenaki* and *kartacze* were declared tourist attractions with the highest values of classification function. In both cases, at *p* = 0.043 and *p* = 0.010, respectively, such declarations were made by tourists surveyed in Poland. At *p* = 0.008 and *p* ≤ 0.001, respectively, *kołduny* and *kiszka ziemniaczana* were more important tourist attractions for respondents in Lithuania, and only *kołduny* was a dish of importance for the region’s promotion. Regional distinctiveness was associated with *kiszka ziemniaczana*, *kołduny* and *czenaki*. At *p* = 0.050 and *p* = 0.041, respectively, *kiszka ziemniaczana* and *czenaki* had higher values of classification function for respondents in Lithuania (Table 8).

## 5. Discussion

The research objectives of the work reported here were fully attained. It was demonstrated that an attractiveness of regional dishes, in particular their taste- and smell-associated appeal, affected the purchasing decisions of consumers and the region’s promotion and clearly accentuated its regional distinctiveness. The hypothetic assumptions were confirmed, namely, that some traditional dishes of Polish-Lithuanian cuisine may be a tourist attraction of the region, can contribute to its promotion and, as a result, increase tourists’ visits to the area.

Culinary tourism provides social and cultural benefits for the inhabitants who, through local culinary products, can cultivate their cultural belongingness [21], as well as provide social and economic benefits for the locality by, e.g., increasing the opportunities of local employment [57]. The cultural dimension defines regional cuisine as cuisine rooted in the landscape which is affected by local traditions and other intangible elements [58,59].

The validity of choosing six basic traditional dishes of the area on both sides of the Polish-Lithuanian border was also confirmed. Glica [60] pointed to the following traditional dishes in the study area: *kartacze*, *sękacz* (*tree cake*), *karmuszka* (*stew-like soup*) and *cepeliny*, whereas Omieciuch [46] and Woźniczko and Orłowski [61] mentioned *krupniok* (*blood sausage*), *kołacz* (*kalach*), *sękacz* and *babka ziemniaczana* as regional dishes of the border area.

According to Czarniecka-Skubina [62], tourists who get to know characteristic products and dishes and their preparation methods are provided with an opportunity to come into direct contact with the local culture. The present study demonstrated that dishes of the area on both sides of the Polish-Lithuanian border, in particular *kiszka ziemniaczana*, *kołduny*, *soczewiaki* and *chłodnik litewski*, may provide such an opportunity, which is also a confirmation of the present work’s hypothetical assumptions. Cooper and Hall [63] believe that sampling of regional specialities and participation in their production contribute to an expansion of tourists’ knowledge of the history, customs and rituals associated with their consumption as well as the recipes. All in all, it is a form of immersing oneself in other cultures and customs [64]. The study discussed here offered evidence that the aforementioned traditional dishes representing Polish-Lithuanian cuisine are indicative of the regional distinctiveness of the area on both sides of the border; this is true to the greatest extent for *kiszka ziemniaczana*, *kołduny* and *czenaki*. Marketing activities may be utilised to promote and identify local gastronomic products by suggesting the destinations to tourists [65,66]. Björk and Kauppinen-Räisänen [67] found that local food has great potential for attracting tourists and contributing to the overall tourist experience, which in turn indicates marketing potential for the hotel and gastronomic industry.

Research by Kivela and Crotts [68] confirmed that cultural tourism serves to promote a region, in particular its recognition and popularity of local specialities on a national and international scale. Traditional dishes strengthen the effect of the region’s perceived value, and tourists often want to repeat the pleasant experience [69]. Hence, it seems rational for the managers of a tourist destination to promote the region using culinary dishes, as they are not only a factor which attracts first-time visitors but a tool for motivating revisits to the destination motivated by flavour-related memories. The authors of the present work have demonstrated that regional products such as *kartacze*, *czenaki*, *kołduny* and *kiszka ziemniaczana* are the main culinary tourist attractions of the Polish-Lithuanian border areas, with *kołduny* having the additional potential of contributing to the region’s promotion.

According to Kim et al. [10], consumption by tourists of previously unknown local dishes is one of more exciting experiences in tourist destinations. Moreover, Bitušíková [70] found that regional dishes are often sold during fairs and festivals which strengthen the identity of the locals, evoke feelings of solidarity and pride, are a means of restoring cohesion and bonds within the local community and promote the region. Similar conclusions were drawn by Teodoroiu and Tudorache [71], who claim that fairs and festivals are the most important events promoting the region’s intangible culture and culinary heritage. In addition, Hall and Sharples [72] believe that such venues provide an interesting type of entertainment, propagating culinary art as well as cultural and tourist events. The study reported here has demonstrated that regional dishes are the most frequent culinary choices during fairs, festivals, conferences and business meetings, and the analysed dishes of the Polish-Lithuanian border areas are very popular during such events, which seems to indicate that the dishes are the core menu of the people living in the area. Consumption of regional dishes appears to be a less popular decision in restaurants. Encouraging consumption of regional dishes in restaurants is of importance for the future of the region because a large number of tourists explore destinations through cuisine and are inclined to spend a substantial part of a trip’s budget on food [73]. The average tourist has been shown to spend 30% [6], and in some cases over 40% [74,75], of their total trip expenditure on consumption in restaurants.

Mazurek-Kusiak et al. [76] pointed to the flavour of products as the most significant factor contributing to purchases of regional dishes. Taste is the predominant means of tourists’ familiarising themselves with traditions and customs of the place they are visiting, with originality being the second most important factor. Consumers become interested in the product they cannot purchase in other regions of Poland or Lithuania. Through such products, they are given an opportunity to learn about the culture, culinary heritage and tradition of the localities they visit. Also, smell is a factor easily attracting consumers to culinary products because it stimulates taste. An appealing aroma adds to the prestige and aesthetics of the product and immerses customers in a pleasurable atmosphere. The appearance tended to be of the least importance. Similar findings were reported by Warmińska et al. [34], Fox [77] and Kivela and Crotts [68], and they were also confirmed in the present study including tourists visiting an area on both sides of the Polish-Lithuanian border.

It has been increasingly popular in the global tourist environment to include local food in the offerings of many tourist destinations [67,78,79]. Locality is often associated with sustainable tourism development, which stresses regional identity and protection as a core of the destination’s competitiveness [12,21]. Local cuisine has become an important merit in the marketing of a destination, and food has developed into a motivation to travel [10] and one of major factors affecting the final decision of which destination to visit [65,80,81].

## 6. Conclusions

Traditional regional dishes of Polish-Lithuanian border cuisine are the most frequently consumed during fairs, festivals, conferences and business meetings, which is probably due to the fact that tourists stay in this area mainly for the purpose of participating in group events such as fairs, regional festivities or work-related meetings. Restaurants are definitely a less popular choice for tourists visiting both Poland and Lithuania. It is a challenge for restaurant owners to encourage tourists to often visit their establishments where they might choose from a variety of local dishes.

Tourists want to explore a given region by tasting local cuisine; they are in search of unique original products while paying much attention to the smell and taste of the dishes they consume. The product’s appearance is of less significance while making purchasing or consumption decisions.

Flavour-related experiences associated with and preferences for individual dishes representing Polish-Lithuanian cuisine were very similar for both groups of respondents. It indicates there is a possibility of establishing cooperation to promote the products in the study area and thus enhance its tourist appeal.

The results of the work confirmed the hypothesis assuming that some traditional dishes representing the Polish-Lithuanian cuisine of the region have the potential to become a tourist attraction of the region and, by contributing to its promotion, encourage tourists to visit the area.

The findings of the present work agree with previous results of analyses pertaining to regional cuisines of various areas. It confirms that, by promoting the cuisine of the Polish-Lithuanian border area, the development of gastronomy including regional dishes may contribute to an increase in tourist movement and that individual dishes may be region-specific products.

As a rule, regional products are produced from organic raw materials following the local custom. Open borders and freedom of movement assist in tourism development, which allows visitors to be better acquainted with regions, particularly those that are less popular and rarely visited by tourists. The present study may contribute to an increase in the popularity and promotion of local products in a given region, thus improving the economic situation of these areas.

## Figures and Tables

**Table 1:** Consumption frequency of and familiarity with dishes representing Polish-Lithuanian cuisine among tourists visiting areas along the Polish-Lithuanian border.

Dish Type	Tourists in Poland		Tourists in Lithuania		*t* Test Value	*p* Value
	$\bar{x}\;\pm \;SD$	n	$\bar{x}\;\pm \;SD$	n		
*kartacze*	3.37 ± 1.33	383	3.47 ± 1.32	376	1.355	0.175
*kiszka ziemniaczana*	4.30 ± 1.26	383	4.98 ± 1.12	376	12.872	0.001 *
*soczewiaki*	3.43 ± 1.43	383	3.02 ± 1.40	376	5.339	0.001 *
*kołduny*	3.38 ± 1.29	383	3.39 ± 1.22	376	0.047	0.964
*czenaki*	3.76 ± 1.33	383	3.72 ± 1.24	376	0.646	0.517
*chłodnik litewski*	3.80 ± 1.33	383	3.81 ± 1.26	376	0.216	0.828

* Level of significance at *p* ≤ 0.05.

**Table 2:** The role of consumption of and familiarity with dishes representing Polish-Lithuanian cuisine among tourists visiting areas along the Polish-Lithuanian border.

Dish Type	Wilks’ Lambda: 0.539 F = 11.907 *p* < 0.001 *			Classification Function	
	Wilks’ Lambda	F Value	*p* Value	Tourists in Poland	Tourists in Lithuania
*kiszka ziemniaczana*	0.537	299.23	0.001 *	4.779	6.059
*kołduny*	0.561	1.803	0.178	1.553	1.605
*soczewiaki*	0.554	89.62	0.001 *	0.146	0.310
*chłodnik litewski*	0.582	17.08	0.001 *	0.271	0.035
Constant				14.336	18.164

* Level of significance at *p* ≤ 0.05.

**Table 3:** Popularity of places of consumption of dishes representing Polish-Lithuanian cuisine among tourists visiting areas along the Polish-Lithuanian border.

Dish Type and Consumption Place	Tourists in Poland		Tourists in Lithuania		*t* Test Value	*p* Value
	$\bar{x}\;\;\pm \;SD$	n	$\bar{x}\;\pm \;SD$	n		
*kartacze*—restaurants	3.20 ± 1.52	383	3.24 ± 1.42	376	0.457	0.647
*kartacze*—fairs	3.25 ± 1.45	383	3.20 ± 1.34	376	0.687	0.497
*kartacze*—conferences	2.77 ± 1.20	383	2.78 ± 1.18	376	0.183	0.850
*kiszka ziemniaczana*—restaurants	3..26 ± 1.35	183	3.18 ± 1.35	376	0.998	0.318
*kiszka ziemniaczana*—fairs	1.91 ± 1.34	383	1.77 ± 1.23	376	1.835	0.667
*kiszka*—conferences	2.65 ± 1.38	383	2.66 ± 1.33	376	0.206	0.836
*soczewiaki*—restaurants	3.56 ± 1.53	383	3.62 ± 1.44	376	0.756	0.449
*soczewiaki*—fairs	2.38 ± 1.49	383	2.61 ± 1.49	376	2.790	0.005 *
*soczewiaki*—conferences	3.13 ± 1.51	383	3.29 ± 1.37	376	1.958	0.050 *
*kołduny*—restaurants	2.19 ± 1.43	383	2.31 ± 1.44	376	1.450	0.147
*kołduny*—fairs	2.54 ± 1.46	383	2.74 ± 1.38	376	2.487	0.013 *
*kołduny*—conferences	2.00 ± 1.35	383	2.21 ± 1.33	376	2.630	0.008 *
*czenaki*—restaurants	2.57 ± 1.51	383	2.64 ± 1.39	376	0.894	0.372
*czenaki*—fairs	2.13 ± 1.36	383	2.33 ± 1.39	376	2.607	0.009 *
*czenaki*—conferences	2.01 ± 1.31	383	1.99 ± 1.26	376	0.227	0.825
*chłodnik litewski*—restaurants	1.62 ± 1.13	383	1.68 ± 1.12	376	0.849	0.395
*chłodnik litewski*—fairs	1.48 ± 1.02	383	1.62 ± 1.18	376	3.265	0.001*
*chłodnik litewski*—conferences	3.34 ± 1.29	383	3.47 ± 1.22	376	1.744	0.813

* Level of significance at *p* ≤ 0.05.

**Table 4:** The importance of the place of consumption of dishes representing Polish-Lithuanian cuisine for tourists visiting areas along the Polish-Lithuanian border.

Dish Type and Consumption Place	Wilks’ Lambda: 0.539 F = 11.927 *p* < 0.001 *			Classification Function	
	Wilks’ Lambda	F Value	*p* Value	Tourists in Poland	Touristsin Lithuania
*kiszka ziemniaczana*—fairs/festivals	0.512	9.948	0.002 *	3.767	3.586
*kartacze*—fairs/festivals	0.531	3.823	0.050 *	2.477	2.879
*chłodnik litewski*—conferences/business meetings	0.522	3.205	0.074	2.103	2.190
*kiszka ziemniaczana*—restaurants	0.512	4.745	0.029 *	1.343	1.242
*kołduny*—fairs/festivals	0.529	2.369	0.124	0.944	1.030
*soczewiaki*—fairs/festivals	0.548	1.650	0.199	0.846	0.899
*chłodnik litewski*—fairs/festivals	0.564	12.390	0.001 *	0.641	0.424
*czenaki*—conferences/business meetings	0.517	1.480	0.228	0.438	0.377
Constant				22.185	22.455

* Level of significance at *p* ≤ 0.05.

**Table 5:** Evaluation of attributes of dishes representing Polish-Lithuanian cuisine by tourists visiting areas along the Polish-Lithuanian border.

Dish Type	Tourists in Poland		Tourists in Lithuania		*t* Test Value	*p* Value
	$\bar{x}\;\pm \;SD$	n	$\bar{x}\;\;\pm \;SD$	n		
*kartacze*—appearance	3.39± 1.63	383	3.29 ± 1.53	376	1.172	0.241
*kartacze*—flavour	1.84 ± 1.26	383	2.09 ± 1.31	376	3.444	0.001 *
*kartacze*—smell	1.95 ± 1.35	383	2.27 ± 1.40	376	4.078	0.001 *
*kiszka ziemniaczana*—appearance	1.80 ± 1.23	383	1.81 ± 1.21	376	0.138	0.890
*kiszka ziemniaczana*—flavour	1.81 ± 1.29	383	1.96 ± 1.34	376	2.057	0.040 *
*kiszka ziemniaczana*—smell	2.19 ± 1.43	383	2.30 ± 1.44	376	1.408	0.159
*soczewiaki*—appearance	1.96 ± 1.40	383	2.20 ± 1.39	376	3.102	0.002 *
*soczewiaki*—flavour	2.47 ± 1.43	383	2.44 ± 1.51	376	1.089	0.376
*soczewiaki*—smell	1.82 ± 1.30	383	1.91 ± 1.28	376	1.042	0.297
*kołduny*—appearance	3.10 ± 1.52	383	3.15 ± 1.47	376	0.567	0.573
*kołduny*—flavour	2.54 ± 1.46	383	2.71 ± 1.39	376	2.046	0.040*
*kołduny*—smell	3.97 ± 1.08	383	3.72 ± 1.19	376	3.948	0.001*
*czenaki*—appearance	2.33 ± 1.25	383	2.51 ± 1.26	376	2.556	0.010*
*czenaki*—flavour	2.99 ± 1.11	383	3.11 ± 1.13	376	1.849	0.064
*czenaki*—smell	2.80 ± 1.28	383	2.19 ± 1.31	376	0.865	0.386
*chłodnik litewski*—appearance	2.13 ± 1.28	383	2.19 ± 1.12	376	0.865	0.386
*chłodnik litewski*—flavour	2.93 ± 1.45	383	3.13 ± 1.42	376	2.507	0.012 *
*chłodnik litewski*—smell	2.32 ± 1.40	383	2.46 ± 1.38	376	1.899	0.058

* Level of significance at *p* ≤ 0.05.

**Table 6:** Attributes of dishes representing Polish-Lithuanian cuisine distinguished by tourists visiting areas along the Polish-Lithuanian border.

Dish Type	Wilks’ lambda: 0.487 F = 19.912 *p* < 0.001 *			Classification Function	
	Wilks’ Lambda	F Value	*p* Value	Tourists in Poland	Tourists in Lithuania
*kołduny*—smell	0.487	7.429	0.001 *	5.028	5.876
*soczewiaki*—flavour	0.497	6.287	0.001 *	5.423	5.012
*chłodnik litewski*—flavour	0.452	1.976	0.132	2.799	2.861
*kartacze*—appearance	0.489	3.221	0.027 *	2.342	2.098
*chłodnik litewski*—smell	0.508	2.878	0.057	1.704	1.658
*kołduny*—flavour	0.467	2.978	0.048 *	1.136	1.018
*kołduny*—appearance	0.518	1.238	0.287	0.741	0.688
*czenaki*—flavour	0.502	3.457	0.018 *	0.089	0.163
Constant				19.717	19.817

* Level of significance at *p* ≤ 0.05.

**Table 7:** Assessment of the effect of dishes representing Polish-Lithuanian cuisine on the region’s development by tourists visiting areas along the Polish-Lithuanian border.

Dish Type	Tourists in Poland		Tourists in Lithuania		*t* Test Value	*p* Value
	$\bar{x}\;\pm \;SD$	n	$\bar{x}\;\;\pm \;SD$	n		
*kartacze*—region’s promotion	1.93± 1.35	383	2.07 ± 1.33	376	1.785	0.074
*kartacze*—regional distinctiveness	2.57 ± 1.46	383	2.62 ± 1.42	376	0.650	0.510
*kartacze*—tourist attraction	4.03 ± 1.09	383	3.76 ± 1.10	376	3.747	0.001 *
*kiszka ziemniaczana*—region’s promotion	1.70 ± 1.25	383	1.73 ± 1.22	376	0.361	0.717
*kiszka ziemniaczana*—regional distinctiveness	2.71 ± 1.48	383	3.06 ± 1.44	376	4.143	0.001 *
*kiszka ziemniaczana*—tourist attraction	2.48 ± 1.52	383	2.57 ± 1.46	376	0.744	0.373
*soczewiaki*—region’s promotion	1.53 ± 1.10	383	1.69 ± 1.11	376	2.424	0.015 *
*soczewiaki*—regional distinctiveness	1.55 ± 1.13	383	1.60 ± 1.13	376	0.836	0.404
*soczewiaki*—tourist attraction	1.93 ± 1.32	383	2.02 ± 1.29	376	1.007	0.313
*kołduny*—region’s promotion	3.42 ± 1.66	383	3.27 ± 1.58	376	1.634	0.102
*kołduny*—regional distinctiveness	3.21 ± 1.42	383	3.30 ± 1.37	376	1.088	0.276
*kołduny*—tourist attraction	2.61 ± 1.50	383	2.90 ± 1.47	376	3.384	0.002 *
*czenaki*—region’s promotion	2.85 ± 1.53	383	3.04 ± 1.49	376	2.249	0.024 *
*czenaki*—regional distinctiveness	3.56 ± 1.53	383	3.62 ± 1.48	376	0.756	0.449
*czenaki*—tourist attraction	4.33 ± 0.97	383	4.16 ± 1.05	376	3.070	0.002 *
*chłodnik litewski*—region’s promotion	1.704 ± 1.25	383	1.73 ± 1.19	376	0.367	0.717
*chłodnik litewski*—regional distinctiveness	2.71 ± 1.47	383	3.06 ± 1.44	376	4.143	0.001 *
*chłodnik litewski*—tourist attraction	2.18 ± 1.52	383	2.57 ± 1.44	376	4.382	0.001 *

* Level of significance at *p* ≤ 0.05.

**Table 8:** Evaluation by tourists of Polish-Lithuanian cuisine attractiveness and its effect on the border region’s promotion.

Dish Type	Wilks’ Lambda: 0.589 F = 13.968 *p* < 0.001 *			Classification Function	
	Wilks’ Lambda	F Value	*p* Value	Tourists in Poland	Tourists in Lithuania
*czenaki*—tourist attraction	0.612	4.087	0.043 *	3.953	3.831
*kartacze*—tourist attraction	0.593	6.567	0.010 *	2.548	2.412
*kołduny*—region’s promotion	0.621	3.262	0.070	1.411	1.347
*kiszka ziemniaczana*—regional distinctiveness	0.616	3.634	0.050 *	1.156	1.239
*kołduny*—tourist attraction	0.598	7.059	0.008 *	0.631	0.714
*kiszka ziemniaczana*—tourist attraction	0.576	15.897	0.001 *	0.525	0.688
*kołduny*—regional distinctiveness	0.602	3.542	0.086	0.383	0.412
*czenaki*—regional distinctiveness	0.618	4.090	0.041 *	0.267	0.289
Constant				23.367	23.445

* Level of significance at *p* ≤ 0.05.

## Data Availability

Data is contained within the article.
